# Nationwide Temporal Dynamics of Mammal Communities Across South Korea: Dominance Shifts and Predator—Prey Implications

**DOI:** 10.3390/ani15233441

**Published:** 2025-11-28

**Authors:** Taewoo Yi, Tae Gwan Kim, Bae Keun Lee, Sol Park, Jongchul Park, Junseok Lee

**Affiliations:** 1 Team of National Ecosystem Survey, National Institute of Ecology, Seochon-gun 33657, Republic of Korea; ytw117@nie.re.kr (T.Y.); animal@nie.re.kr (B.K.L.); qkrthf96@nie.re.kr (S.P.); 2 Department of Microbiology, Pusan National University, Busan 46241, Republic of Korea; tkim@pusan.ac.kr; 3 Department of Geography, Kongju National University, Gongju-si 32588, Republic of Korea; jcp@kongju.ac.kr

**Keywords:** national ecosystem survey, mammal community, dominant species, apex predator absence, conservation management

## Abstract

Understanding long-term changes in mammal communities is essential for evaluating ecosystem dynamics under large-scale environmental transformation. This study analyzed nationwide data from the 3rd (2006–2013), 4th (2014–2018), and 5th (2019–2023) National Ecosystem Surveys to assess temporal shifts in the abundance and dominance structure of medium- and large-sized mammals across the Korean Peninsula. Using standardized survey data, we compared changes in detection frequency, dominance rank, and diversity indices among survey phases. Results revealed a significant restructuring of dominance from the 3rd to 4th survey, followed by stabilization in the 5th phase, indicating a gradual simplification of community structure. The water deer remained the most dominant species throughout all surveys, while the wild boar showed a consistent increase in relative frequency. In contrast, smaller or specialist species such as the Korean hare exhibited notable declines. These patterns correspond to ecological changes driven by forest recovery and the absence of apex predators, which have facilitated the proliferation of adaptable generalist species. The findings suggest that ongoing habitat recovery, though beneficial for biomass and productivity, may simultaneously reduce functional diversity and community balance. This study highlights the value of long-term, standardized monitoring for identifying structural transitions in wildlife communities and understanding their broader implications for biodiversity conservation on the Korean Peninsula.

## Simple Summary

This study analyzed long-term changes in mammal communities across the Korean Peninsula using data from the 3rd (2006–2013), 4th (2014–2018), and 5th (2019–2023) National Ecosystem Surveys. Results showed that a few adaptable species—particularly the water deer (*Hydropotes inermis*) and wild boar (*Sus scrofa*)—have become increasingly dominant, while smaller or specialist species such as the Korean hare (*Lepus coreanus*) have declined. These shifts are closely linked to forest recovery, human land-use changes, and the absence of top predators like the tiger (*Panthera tigris*). As forests recovered after decades of deforestation, large herbivores and omnivores benefited from improved habitat conditions. However, the observed dominance changes should be interpreted cautiously, as they may reflect both ecological shifts and survey methodology improvements. Over time, this has led to a simplified and unbalanced community structure, where a few generalist species dominate while overall diversity and evenness decline. The findings highlight the need for continued ecological monitoring and a better understanding of how habitat restoration and predator loss interact to shape wildlife communities on the Korean Peninsula.

## 1. Introduction

Since the Korean War (1950–1953), forests on the Korean Peninsula have undergone profound transformations, evolving from severe devastation to the current stage of qualitative management. In the immediate post-war decades of the 1950s and 1960s, extensive logging during military activities, excessive timber harvesting for reconstruction, and surging demand for fuelwood left large areas of mountains barren. In the 1970s and 1980s, nationwide reforestation programs rapidly established monoculture plantations dominated by species such as *Pinus rigida* and *Robinia pseudoacacia*, which successfully achieved short-term greening of degraded landscapes [1]. Over time, natural succession introduced broad-leaved species and facilitated the development of mixed forests, resulting in increasingly complex vertical structures and the recovery of food resources and habitat heterogeneity [2]. These ecological improvements provided the foundation for recolonization by birds, insects, and mammals. Consequently, Korean forests transitioned from simple green cover to multifunctional ecosystems that support both biodiversity and ecological processes [3].

Mammal communities are subject to continuous spatiotemporal fluctuations under the combined influence of forest structure, climatic conditions, prey distribution, land-use change, and anthropogenic disturbances [4,5,6]. On the Korean Peninsula, where human population density is high and land-use conversion is frequent, forest restoration, farmland decline, urban expansion, and species management have simultaneously shaped mammalian community composition and interaction networks [7,8]. These changes extend beyond the ranges and population sizes of individual species, influencing species diversity, evenness, functional guild balance, and food-web stability.

The long-term restructuring of mammal communities is also closely tied to historical events. Intense hunting pressure from the late Joseon dynasty through the Japanese colonial period led to the extirpation of apex predators such as the tiger by the 1940s, while the leopard (*Panthera pardus*) persisted only until the latter half of the 20th century. During the Korean War, widespread forest destruction and anthropogenic disturbances further reduced mammal habitats, triggering population collapses and severe ecological disruption. These historical events provide essential context for interpreting long-term mammalian community dynamics on the Korean Peninsula.

As a result, forest recovery and ecosystem restoration progressed in the absence of apex predators, a condition that likely disrupted the structural balance of the food web and led to the relative expansion of mesopredators as well as large-bodied herbivores and omnivores [9,10,11].

The National Ecosystem Survey, conducted over the past three decades, represents the only nationwide monitoring system capable of tracking these long-term changes. However, its application has largely been restricted to regional reports or short-term analyses of individual species, with few attempts to integrate temporal trends across survey phases. Consequently, understanding of long-term mammalian community restructuring in Korea remains limited.

To address this gap, the present study analyzes data from the 3rd (2006–2013), 4th (2014–2018), and 5th (2019–2023) National Ecosystem Surveys to examine the long-term dynamics of mammal communities on the Korean Peninsula. Specifically, we aim to (i) evaluate rank-order shifts and restructuring of dominance hierarchies, (ii) quantify temporal variations in species diversity and evenness, and (iii) interpret the underlying ecological drivers in the context of habitat structure, prey resource distribution, and the absence of apex predators.

This study represents the first attempt to comprehensively integrate nationwide, long-term monitoring data of Korean mammals. Its findings provide essential scientific evidence for biodiversity conservation strategies, habitat management, and climate change adaptation at the national scale.

## 2. Materials and Methods

### 2.1. Mammal Survey Methods in the National Ecosystem Survey

**Figure 1:**
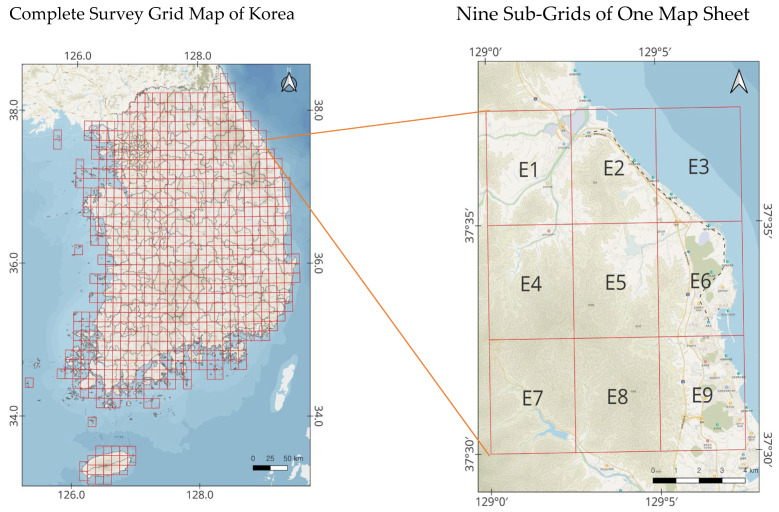
Survey units of the National Ecosystem Survey (map sheets and grids). E1–E9 indicate the nine sub-grids within a 1:25,000 map sheet.

The mammal data used in this study were collected in accordance with the standardized protocols of the National Ecosystem Survey, conducted under the supervision of the Ministry of Environment. The survey covered the entire Korean Peninsula, excluding marine areas, and the basic survey unit was a 1:25,000-scale topographic map sheet. Each 1:25,000-scale map sheet (approximately 12 km in width and 13 km in height) was subdivided into nine rectangular sub-grids (4 × 4.3 km). From each map sheet, two sub-grids were selected using a stratified random approach to ensure both representativeness and inclusion of habitats with high predicted biodiversity. The selection process prioritized ecological heterogeneity (forest type, elevation, and proximity to water) while maintaining spatial randomness. Within each selected sub-grid, trained field surveyors conducted standardized transect surveys for approximately 4–6 h per day over two consecutive days, recording both direct observations (visual sightings) and indirect evidence (tracks, feces, burrows, and carcasses). In later survey phases, infrared-triggered camera traps were deployed at sites of high mammal activity such as game trails and riparian zones, and operated continuously for approximately three months per grid. Figure 1 illustrates the spatial layout of these 4 × 4.3 km sub-grids and their spatial distribution across South Korea. Table 1 summarizes the survey effort, including the number of surveyed map sheets, total sampled grids, and cumulative sampling area for each NES phase. This semi-random, effort-standardized sampling framework follows the National Ecosystem Survey Guidelines for Terrestrial Mammals (Ministry of Environment & National Institute of Ecology, 2021). It ensures comparability across survey periods while minimizing observer bias and providing statistically robust coverage of mammal habitats across varying landscape types.

The National Ecosystem Survey used a transect-based design combining direct observations and automated monitoring to maximize species detection probability. Surveys of medium- and large-sized mammals were conducted on foot, recording both direct sightings and indirect signs such as tracks, feces, claw marks, and other traces. Detected signs were photographed and measured (length and width) in the field, and species were identified on site whenever possible. When species identification was uncertain, samples such as hair or feces were collected and later verified through laboratory analysis. For morphologically similar species (e.g., leopard cat (*Prionailurus bengalensis*) vs. domestic cat (*Felis catus*), Siberian roe deer (*Capreolus pygargus*) vs. water deer), multiple lines of evidence were integrated to ensure accurate identification. In cases where identification was ambiguous, fecal and other field samples were brought to the laboratory for detailed examination of morphological characteristics and contents, while molecular techniques were not employed.

For medium- and large-sized mammals that are difficult to observe during daytime, infrared-triggered camera traps were deployed. At least one camera was installed per map sheet grid, with placement at locations of high likelihood of occurrence, such as animal trails, riparian zones, and habitat edges. Cameras (Browning BTC-6DCLN) were installed at each site for three months and were regularly inspected for maintenance and data retrieval. They were typically mounted at a height of 50 cm to 1 m above the ground. Image records were subsequently classified by species based on identifiable morphological traits [12].

### 2.2. Analytical Methods for Mammal Observation Frequency

This study analyzed temporal changes in mammal communities using a total of 186,661 survey records collected during the 3rd (2006–2013; 66,621 records), 4th (2014–2018; 42,662 records), and 5th (2019–2023; 77,378 records) National Ecosystem Surveys. In these surveys, the basic unit of mammal investigation was the topographic map sheet, within which multiple methods—including track surveys, camera-trap monitoring, and live-trapping—were employed simultaneously (Figure 1).

Table 1 summarizes the survey methods applied in each phase. Although the overall methodology remained consistent across the 3rd, 4th, and 5th National Ecosystem Surveys, camera traps were first introduced in the 4th survey, enhancing detection accuracy. In the 5th survey, the number of cameras per map sheet was increased to two, representing the most notable methodological improvement.

Because the same individual could be repeatedly detected within a single grid by different survey methods (e.g., camera trap, track, or trap capture), frequency analysis was standardized by treating all detections of the same species within a grid as a single observation. This approach minimized redundancy and ensured consistency in calculating observation frequency.

Using these standardized species frequencies, we conducted time-series analyses to assess rank-order shifts and dominance restructuring across survey phases. Relative occurrence rates were calculated for each species within each survey phase, and these values were used to determine species ranks. In particular, rank-transition analyses were performed for the top 15 species, and temporal changes in species ranks and dominance patterns were visualized using Sankey diagrams to track the flow of rank dynamics over time.

### 2.3. Statistical Analyses

All statistical analyses were performed using nonparametric methods to account for non-normal data distributions and unequal variances.

Temporal and interspecific changes in species composition and relative abundance were assessed using Spearman’s rank correlation coefficient (ρ) and Kendall’s rank correlation (τ) to evaluate the strength and direction of monotonic relationships.

All statistical analyses were conducted using non-parametric methods to accommodate non-normal data distributions and unequal variances. Differences among survey phases were assessed using the Kruskal–Wallis test, followed by Dunn–Holm post hoc comparisons for pairwise evaluations. Community-level differences were tested using PERMANOVA (Permutational Multivariate Analysis of Variance) based on the Bray–Curtis dissimilarity matrix, and PERMDISP was applied to verify homogeneity of dispersion. Additionally, bias-corrected and accelerated (BCa) 95% bootstrap confidence intervals were computed to evaluate estimate precision. All analyses were performed in R version 4.5.1, using the vegan package.

### 2.4. Diversity Indices and Evenness

Species diversity and the evenness of individual distribution were analyzed for each survey phase. To evaluate species diversity, we applied the Shannon–Wiener diversity index (*H′*) and the Simpson index, and further assessed community evenness using the corresponding evenness index [13,14].

Shannon–Wiener diversity index (*H′*):

(1) $$H^{\prime }=-\sum_{i=1}^{S}p_{i}\ln\left(p_{i}\right)$$

Simpson index:

(2) $$D=1-\sum \left(p_{i}^{2}\right)$$

Here, pi represents the relative abundance of species *i*, calculated as the number of individuals of species *i* divided by the total number of individuals.

Evenness index:

(3) $$E=\frac{H^{\prime }}{\ln\left(S\right)}$$

where *S* is the total number of species.

### 2.5. Analysis of Beta Diversity

Beta diversity is an index that measures the degree of variation in species diversity across different temporal or environmental contexts. In this study, beta diversity was calculated based on changes in species-specific observation proportions. This approach follows the conceptual framework of Whittaker [15,16] and the extrapolative estimation principles proposed by Colwell and Coddington [17], which provide a basis for assessing species richness and community turnover from finite sampling data.

(4) $$\beta =\frac{\sum_{i=1}^{n}\left|\left.S_{i}-S_{j}\right|\right.}{n}$$

Here, *Si* and *Sj* represent the proportional species composition in surveys *i* and *j*, respectively. By applying this formula, beta diversity values were derived to quantify compositional differences among the survey phases.

A PERMANOVA (Permutational Multivariate Analysis of Variance) and a PERMDISP [18] were conducted to test differences in community composition and homogeneity among survey phases. The analysis was based on a Bray–Curtis dissimilarity matrix with 999 permutations to assess significance. These non-parametric methods followed [18,19]. All analyses were performed using the adonis2 function in the R version 4.5.1 with the vegan package.

## 3. Results

This study compared and analyzed changes in observation frequency and dominance rank of mammal species using data from the 3rd, 4th, and 5th National Ecosystem Surveys. As the surveys were conducted under standardized frameworks and methodologies, they provide long-term, nationwide ecological datasets that capture mammal distribution and habitat conditions across the Korean Peninsula, independent of specific spatial or temporal constraints. Such datasets not only improve our understanding of population fluctuations over time but also offer essential scientific evidence for identifying long-term ecological trends and informing conservation policies. In particular, rank shifts in conservation-priority or subdominant species serve as key indicators for reassessing conservation priorities and refining management strategies.

Although surveys within the same phase were conducted under consistent conditions for each map sheet, variations in factors such as survey duration and the number of camera traps between phases may have influenced the estimation of species abundance. Moreover, the reliance on camera traps and indirect indicators (e.g., tracks, feces), together with variable detection rates, could further affect the accuracy of abundance estimates, particularly for species that are highly cryptic or occur at low densities.

**Figure 2:**
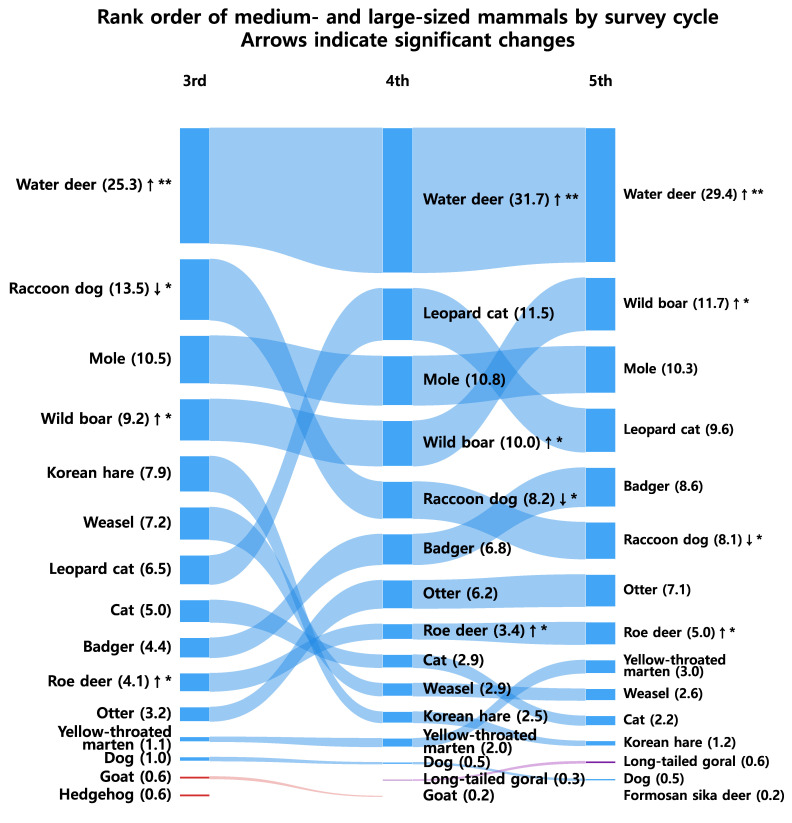
Species with significant changes in relative frequency between survey phases. Arrows (↑ or ↓) indicate the direction of change. A single asterisk (*) denotes a significant difference at *p* < 0.05, and a double asterisk (**) denotes a significant difference at *p* < 0.01 (Holm correction applied).

Figure 2 illustrates rank transitions and shifts in dominance among medium- and large-sized mammals across the survey phases. The water deer remained the most dominant species throughout all surveys, representing 27.0%, 31.7%, and 29.4% of total detections in the 3rd, 4th, and 5th surveys, respectively. The wild boar (*Sus scrofa*) showed a continuous increase, rising from 4th to 2nd place, while the leopard cat also exhibited a notable upward trend.

Collectively, the top five species accounted for 52.9%, 62.9%, and 62.4% of all detections across the three survey phases, indicating an increase in dominance concentration from the 3rd to the 4th survey (*p* < 0.05 in community composition), which remained at a comparably high level without a significant additional increase in the 5th survey (Kruskal–Wallis *p* = 0.245). In contrast, smaller or specialist species such as the Korean hare declined significantly, suggesting a gradual loss of community evenness and increasing structural imbalance. Rank correlation analyses revealed consistently strong monotonic concordance among all survey pairs (*ρ* ≈ 0.9), implying that the relative hierarchical structure of dominant and subordinate species was largely preserved through time (Table A1). The marginally reduced correlation between the 3rd and 5th surveys indicates limited cumulative rank drift over the extended temporal interval.

**Figure 3:**
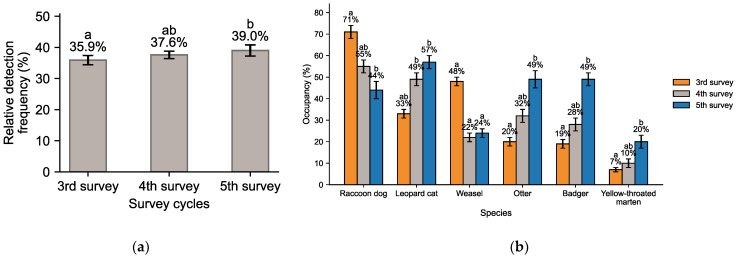
Carnivora (six species): (**a**) Relative detection frequency and (**b**) occupancy ratio (mean ± 95% BCa CI). Different letters indicate significant pairwise differences among survey cycles (Dunn–Holm, *p* < 0.05); bars sharing the same letter are not significantly different, and “ab” indicates an intermediate group.

Figure 3 summarizes temporal changes in carnivore species. The relative frequency of six carnivores (Eurasian otter, leopard cat, raccoon dog, Asian badger, yellow-throated marten, and Siberian weasel) increased from 35.9% (3rd survey) to 37.6% (4th) and 39.0% (5th). Since the 4th survey, their proportion remained consistently above 30%, reflecting a stable increase in the role of carnivores within mammal communities.

Occupancy rates revealed divergent trends among Carnivora. The leopard cat expanded steadily (33% → 49% → 57%), while the otter showed marked growth (20% → 32% → 49%). The badger also increased (19% → 28% → 49%), and the yellow-throated marten rose gradually (7% → 10% → 20%). In contrast, the raccoon dog declined sharply from 71% to 44% before partially recovering to 55%, and the weasel decreased substantially from 48% to 22–24%. Overall, except for raccoon dog and weasel, Carnivora species exhibited continuous expansion, particularly leopard cat, otter, and badger, which showed dramatic increases in occupancy.

**Figure 4:**
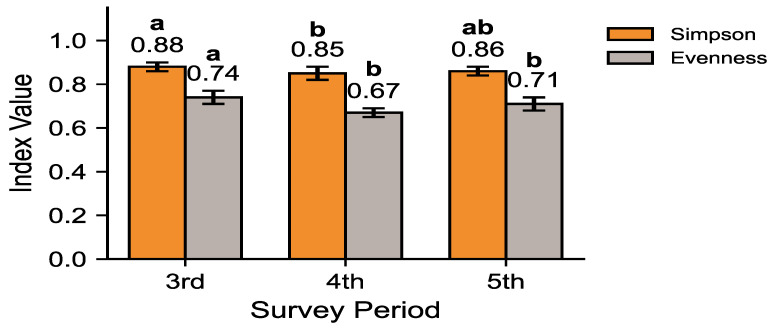
Temporal changes in species dominance and evenness: Simpson index and Evenness index. Bars represent mean ± 95% BCa confidence intervals. Different letters indicate statistically significant pairwise differences between survey phases (Dunn–Holm, *p* < 0.05).

Figure 4 evaluates species dominance and evenness using Simpson and evenness indices. Species diversity, as measured by the Shannon diversity index (H′), was slightly lower in the 4th and 5th surveys (≈2.20–2.25) compared to the 3rd survey (≈2.34), but the Kruskal–Wallis test revealed no significant difference among survey periods (H = 2.85, df = 2, *p* = 0.245). Dunn–Holm post hoc comparisons confirmed the absence of pairwise significance, and the 95% BCa bootstrap confidence intervals overlapped considerably, indicating stable diversity across surveys. In terms of Simpson index values, the 3rd survey showed the lowest dominance (0.88), while the 4th and 5th surveys exhibited slightly higher dominance (0.85 and 0.86, respectively). Evenness was also highest in the 3rd survey (0.74), decreasing to 0.67 and 0.71 in the 4th and 5th surveys. Overall, the 3rd survey represented the most balanced community, while the subsequent surveys reflected increased dominance of a few species and a slight reduction in evenness. These patterns suggest that the community underwent reorganization after the 3rd survey and subsequently entered a more stabilized state.

**Figure 5:**
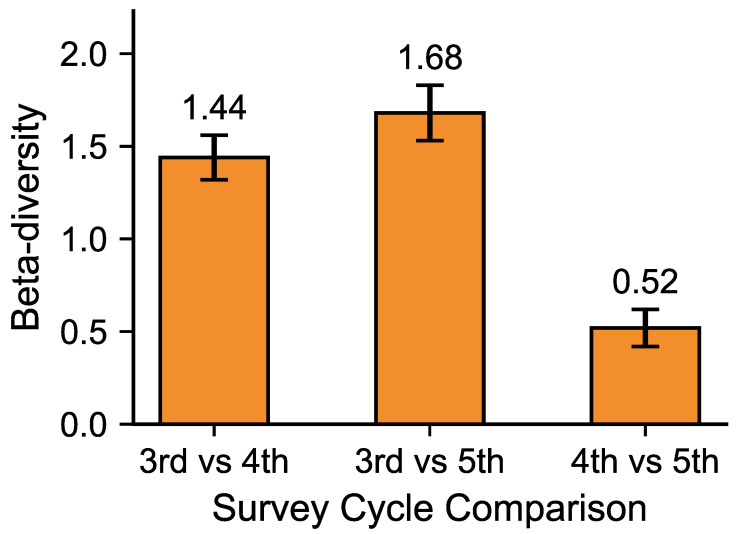
Temporal changes in β-diversity (Bray–Curtis) of mid- to large-sized mammal communities. Bars show mean ± 95% BCa CI. Statistical differences in community composition were tested using PERMANOVA (*p* = 0.021) and homogeneity confirmed by PERMDISP (*p* > 0.05).

Figure 5 shows beta diversity between survey phases. Beta diversity between the 3rd and 4th surveys was 1.44, reflecting significant compositional differences. Between the 3rd and 5th surveys, beta diversity reached its highest value (1.68), indicating major shifts, with diversity and evenness declining while dominance increased. In contrast, beta diversity between the 4th and 5th surveys was only 0.52, suggesting smaller compositional differences and relatively similar community structures. Comparable diversity and evenness indices between these two phases further support the interpretation that they shared similar ecological conditions.

A PERMANOVA [18,19] was conducted to test the statistical significance of differences in community composition among survey phases. The analysis was based on a Bray–Curtis dissimilarity matrix, and 999 permutations were performed to obtain the *p*-value. The results showed that differences in community composition among the survey phases were statistically significant (F = 4.21, *p* = 0.021, R^2^ = 0.68). To verify that these results were not driven by heterogeneous dispersion among groups, a PERMDISP test [18,19] was also performed, confirming homogeneous dispersion (*p* > 0.05). Together, these findings indicate that the observed β-diversity patterns among the 3rd, 4th, and 5th surveys reflected genuine temporal changes in community structure rather than random variation. Corresponding 95% BCa confidence intervals (10,000 bootstrap resamples) are shown in Figure 5.

## 4. Discussion

### 4.1. Dominance Patterns and Community Restructuring

Across all three survey phases, water deer consistently remained the most dominant species, maintaining a clear lead over other taxa. Its persistent top rank reflects its ecological plasticity and adaptability within the recovering ecosystems of the Korean Peninsula. Similarly, the wild boar rose steadily from fourth to second place, illustrating the growing prevalence of generalist species capable of thriving in fragmented and human-modified landscapes. Consistent with previous studies [20,21], dominance of adaptable generalists such as water deer and wild boar has been associated with increased anthropogenic landscapes, confirming the nationwide trend identified in prior NES-based analyses.

Figure 2 illustrates shifts in community dominance structure, showing that the combined proportion of the top five species increased from 52.9% in the 3rd survey to 62.9% and 62.4% in the 4th and 5th surveys, respectively. The marked increase between the 3rd and 4th surveys, followed by stabilization during the 5th, suggests that dominance concentration rose significantly once and then reached a plateau without further significant change. The pattern is consistent with the diversity metrics presented in Figure 4, where the Shannon–Wiener diversity index (H′) and evenness indices declined, while Simpson’s dominance index increased across phases. Collectively, these results demonstrate a reduction in community diversity and evenness, with dominance concentration persisting into the 5th survey (Table A1). Although minor stabilization was observed between the 4th and 5th phases, the overall structure suggests that the mammal community on the Korean Peninsula has not yet returned to a balanced or functionally diverse state.

### 4.2. Ecological Drivers of Dominance Concentration

The increasing dominance of water deer and wild boar observed in this study (Figure 2)—rising from 27.6% to 36.8% in relative detection frequency—likely reflects ecological changes associated with nationwide forest recovery and the long-term absence of apex predators. Since the mid-20th century, reforestation and natural succession have markedly expanded forest cover and increased mature stand proportions [2], creating favorable habitats for generalist herbivores. However, the concurrent decline in Shannon diversity (from 2.34 to 2.20–2.25) and evenness (from 0.74 to 0.67–0.71) (Figure 4) suggests that these structural improvements did not lead to equivalent increases in functional diversity. Thus, while forest recovery provides the environmental context, the compositional shift toward dominance by a few adaptable species represents a measurable reduction in community evenness rather than a generalized biodiversity gain.

At the same time, the absence of apex predators, such as the tiger, removed top-down regulatory control, enabling herbivore and omnivore populations to expand [22,23]. The expansion of agricultural and peri-urban areas further increased the availability of food resources and shelter, favoring species with high behavioral flexibility. Additionally, improvements in survey methodology, such as increased camera trap deployment in the 5th NES, may have influenced detection rates and apparent dominance patterns. Additionally, improvements in survey methodology, such as increased camera trap deployment in the 5th NES, may have influenced detection rates and apparent dominance patterns [24,25,26]. Reflecting these ecological changes, the combined proportion of H. inermis and S. scrofa in total detections rose steadily—from 27.6% in the 3rd survey to 36.4% and 36.8% in the 4th and 5th surveys, respectively. Together, these factors have driven the population expansion and dominance of H. inermis and S. scrofa, consistent with the herbivore release phenomenon observed in other regions following the loss of top predators. Comparable ecological cascades have been widely documented [27], demonstrating that the reduction in large predators such as wolves and cougars in North American forest ecosystems led to increased densities of ungulates, which in turn caused over-browsing and altered forest regeneration patterns. Similarly, ref. [28] described “trophic downgrading” as a global phenomenon, where the removal of apex predators disrupts food-web stability, leading to herbivore overabundance, vegetation degradation, and the simplification of ecosystem structure.

These findings parallel the Korean context, where the loss of apex predators has likely released large herbivores and omnivores from predation pressure, promoting their expansion and dominance. Similar patterns have been reported in regional NES studies [20,21,29,30], reinforcing that dominance restructuring is a nationwide trend across multiple taxa [31,32,33]. While these shifts reflect positive trends in forest recovery and habitat productivity, they also indicate functional imbalance within mammal communities [34,35,36,37]. The rise of a few dominant generalist species, coupled with the decline of subordinate taxa such as the Korean hare, suggests decreasing functional redundancy and ecological resilience [38,39,40,41,42,43,44]. This imbalance may alter vegetation dynamics, interspecific interactions, and trophic stability over time, emphasizing the need for adaptive management that maintains habitat heterogeneity and supports less common taxa.

## 5. Conclusions

This study provides a comprehensive nationwide assessment of long-term restructuring in mammalian communities across South Korea, based on the 3rd (2006–2013), 4th (2014–2018), and 5th (2019–2023) National Ecosystem Surveys (NES). The results demonstrate a progressive concentration of dominance, with adaptable generalist species such as water deer and wild boar increasingly prevailing, while smaller or specialist species, including Korean hare, have steadily declined. The observed trends, including the increase in water deer and wild boar dominance (Figure 2) and the decline in Shannon diversity and evenness (Figure 4), suggest that community composition has become more concentrated over time. These changes likely reflect habitat recovery and predator loss, as documented in previous studies [2,20,21], rather than direct increases in overall biomass or productivity. Our results therefore indicate that although forest restoration has expanded suitable habitats, it has not yet translated into proportional gains in functional or taxonomic diversity. The reduced evenness and dominance concentration observed here point to partial recovery of mammal communities with persistent imbalance in species composition. Thus, beyond quantitative habitat expansion, qualitative aspects—such as maintaining functional diversity, restoring trophic interactions, and conserving habitat heterogeneity—must be integrated into biodiversity management strategies.

From a policy and management perspective, this study underscores the critical role of long-term, standardized monitoring frameworks such as the NES in detecting early signs of community-level transitions and providing scientific evidence for adaptive conservation policy. Future work should integrate environmental drivers such as climate change, land-use dynamics, and anthropogenic pressures to elucidate the mechanisms sustaining dominance concentration. In particular, combining NES datasets with spatial ecological modeling and functional diversity metrics will help clarify causal relationships between habitat restoration and mammal community stability.

In conclusion, mammal communities in Korea appear to be in a transitional stage, reflecting both ecological recovery and functional imbalance. Ensuring long-term stability will require adaptive management strategies that restore trophic balance and sustain multi-level biodiversity. Strengthening continuous, data-driven monitoring under the NES framework will be essential for translating Korea’s forest restoration efforts into sustainable biodiversity recovery and enhanced ecosystem resilience in the decades ahead.

## Figures and Tables

**Table 1:** Summary of mammal survey protocols across the 3rd, 4th, and 5th National Ecosystem Surveys in Korea.

Category	3rd Survey (2006–2013)	4th Survey (2014–2018)	5th Survey (2019–2023)
Survey unit	1:25,000 topographic map sheets divided into nine grids	Same as 3rd survey (map sheet and grid-based unit)	Same as 3rd survey (map sheet and grid-based unit)
Survey period	February–October each year; at least two seasonal surveys	February–October each year; at least two seasonal surveys	February–October each year; at least two seasonal surveys
Survey method	Line-transect surveys by foot; direct and indirect observations	Line-transects combined with camera traps (one camera/map sheet)	Line-transects combined with camera traps (two cam-era/map sheet)
Survey Intensity	At least two grids per map sheet; 10 days	At least two grids per map sheet; 7 days	At least two grids per map sheet; 7 days
Use of camera traps (year of use)	X	O (2016–2018)	O (2019–2023)
Total number of data	66,621	42,662	77,378
Number of survey map (sub grid) sheets	760 (5762)	629 (3681)	746 (5236)

## Data Availability

The data analyzed in this study were derived from the 3rd (2006–2013), 4th (2014–2018), and 5th (2019–2023) National Ecosystem Surveys conducted by the Ministry of Environment of the Republic of Korea. These datasets are managed by the Ministry of Environment and the National Institute of Ecology. While the data are not publicly archived, they may be available upon reasonable request to the relevant institutions. No new datasets were generated by the authors in this study.

## Abbreviations

The following abbreviations are used in this manuscript:

KFS	Korea Forest Service
NES	National Ecosystem Survey
NIE	National Institute of Ecology
RAI	Relative Abundance Index
DMZ	Demilitarized Zone
H′	Shannon–Wiener diversity index
D	Simpson Index
J′	Pielou’s Evenness Index
β	Beta Diversity

## Appendix A

**Table A1:** Statistical summary of temporal community changes.

Category	Test/Model	Metric/Variable	Statistical Result	Interpretation
Rank correlation	Spearman’s ρ	3↔4, 4↔5, 3↔5	3↔4: 0.914, 4↔5: 0.902, 3↔5: 0.873 (*p* < 0.001)	High concordance; structure stabilized after 4th survey
	Kendall’s τ	3↔4, 4↔5, 3↔5	3↔4: 0.772, 4↔5: 0.777, 3↔5: 0.703 (*p* < 0.001)	Confirms rank-shift significance
Diversity indices	Kruskal–Wallis	H, *p*-value	H = 2.85, *p* = 0.245	No significant difference among surveys
	Bootstrap CI (95%)	Shannon index (H’)	3rd = 2.70–2.71, 4th = 2.50–2.52, 5th = 2.50–2.51	Overlapping CIs → Stable diversity
